# Feeding broilers with wheat germ, hops and grape seed extract mixture improves growth performance

**DOI:** 10.3389/fphys.2023.1144997

**Published:** 2023-03-28

**Authors:** Qiangqiang Zou, Weishuang Meng, Chunxiao Li, Tieliang Wang, Desheng Li

**Affiliations:** ^1^ College of Animal Husbandry and Veterinary Medicine, Jinzhou Medical University, Jinzhou, China; ^2^ Anshan Animal Disease Prevention and Control Center, Anshan, China

**Keywords:** wheat germ, hops extract, grape seed extract, broilers, growth performance, blood indicators, faecal microbiota, noxious gas

## Abstract

In the study, Wheat germ, Hops and Grape seed extracts were made into a mixture (BX). The BX was supplemented in AA + broilers diets to investigate the effects of BX on broiler growth performance, blood indicators, microbiota, and noxious gas emissions in faeces. Four hundred and eighty 1-day-old AA + male broilers with an average initial body weight (44.82 ± 0.26) were randomly divided into four dietary treatments of six replicates each, with 20 birds per replicate. The experimental groups consisted of a group fed a basal diet and groups fed basal diet supplemented with 0.05%, 0.1%, and 0.2% BX. The trail was 42 days. The results showed that supplementing the dietary with graded levels of BX linearly increased ADG and ADFI from days 22–42 and 1–42. When dietarys supplemented with 0.2% BX significantly increased ADG and ADFI on days 22–42 and 1–42 (*p* < 0.05). The addition of BX reduced H_2_S and NH_3_ emissions in the faeces; the levels of *E. coli* and *Salmonella* in the faeces were significantly reduced and the levels of *Lactobacillus* were increased (*p* < 0.05). In this trial, when the diet was supplemented with 0.2% BX, faecal levels of *E. coli* and *Salmonella* were consistently at their lowest levels and *Lactobacillus* were at their highest. At the same time, NH_3_ and H_2_S emissions from broiler faecal also had been at their lowest levels. Conclusion: Dietary supplementation with a 0.2% BX could improve the growth performance of broilers and also reduced faecal H_2_S and NH_3_ emissions, as well as faecal levels of *E. coli* and *Salmonella*, and increased levels of *Lactobacillus*. Thus, BX made by Wheat germ, Hops and Grape seed extract is expected to be an alternative to antibiotics. And based on the results of this trial, the recommended dose for use in on-farm production was 0.2%.

## Introduction

A complete grain of wheat consists of bran, endosperm and germ, with the germ making up about 3% of the grain weight (Fardet, 2010). Wheat germ (WG) was the life source of wheat and was a major by-product of the wheat flour industry. WG contains approximately 10%–15% fat, 26%–35% protein, 17% sugar, 1.5%–4.5% fibre, and 4% minerals (Brandolini and Hidalgo, 2012). Matsui et al. (2000) obtained an angiotensin-converting enzyme inhibitory peptide with the sequence Ile-Val-Tyr by isolating a peptide produced by the enzymatic digestion of wheat germ protein. Meanwhile, many researchers had explored the hypolipidemic (Liaqat et al., 2021), anti-aging (Zhao et al., 2021), anti-inflammatory (Sui et al., 2020), and antioxidant effects (Wang et al., 2020) of WG. Studies had shown that broilers fed wheat germ oil achieve higher body weights (Arshad et al., 2013a). When combined with *a*-lipoic acid, wheat germ oil could help improve lipid distribution in broilers (Arshad et al., 2013b). To date, no papers have been published on the effects of crushing wheat germ and feeding it directly to broilers.

Hops (Humulus lupulus) is a perennial plant of the Cannabaceae family, which has only two genera. The genus Humulus and the genus Cannabis. The hops plant plays an important role in human nutrition and culture, as its female inflorescences are used to produce beer. Extracts of the hops plant had an antibacterial effect on Gram-positive bacteria (Teuber and Schmalreck, 1973; Srinivasan et al., 2004). The addition of beta-acids from hops to broiler diets had been reported to improve the overall redox stability and nutritional properties of broilers (Zawadzki et al., 2018). It had been shown that extracts of hops (xanthohumol) could upregulate the expression of phase II enzymes while increasing the protein, activity and glutathione levels of these enzymes (Yao et al., 2015). And, the anti-inflammatory effect of xanthohumol might be related to its modulation of Nrf2-ARE signalling (Lee et al., 2011).

Grape by-products, mainly consisting of skins, seeds and stems, are rich in polyphenols. Grape Seed Extract (GSE) contains mainly proanthocyanidins (PA), catechins, quercetin and tannins. Of these, PA is the most abundant and purest, and is the main substance in the GSE that possesses a biological function. Studies had shown that GSE had antibacterial (Ahn et al., 2004), anti-inflammatory (Carini et al., 2001), antioxidant (Luther et al., 2007), and anticancer (Cheah et al., 2014) activities, with protective effects on host skin (Yamakoshi et al., 2003), cardiovascular (Nunes et al., 2016), liver (Hassan and Al-Rawi, 2013), and nervous system (Mahmoud, 2013). Farahat et al. (2017) reported that GSE increased Newcastle disease virus antibody potency in broiler serum and effectively reduced malondialdehyde levels in meat tissue. In addition, the negative effects of aflatoxin B1 could be effectively reduced by adding a certain amount of GSE to broiler diets (Ali Rajput et al., 2017).

Many studies had reported that the use of botanical preparations in animal feed as dietary supplements could effectively regulate animal metabolism and influence animal welfare and meat quality (Patra and Saxena, 2011; Zawadzki et al., 2017). Many researchers had reported on the physiological properties and beneficial effects of wheat germ, hops and grape seed extracts. However, the effect of adding a mixture of wheat germ, hops and grape seed extract to broiler dietary as a feed additive on broiler production has not been reported. Therefore, in this trial, wheat germ, hops and grape seed extract mixture (BX) was supplemented in AA + broiler diets to study its effects on broiler growth performance, blood indicators, faecal microbiota and noxious gas emissions. It is hoped that this will provide a theoretical basis for the future use of plant additives in poultry production.

## Materials and methods

### Ethics statement

The Animal Conservation and Utilisation Committee of the JZMU approved the animal use agreement.

### Prebiotic sources

BX was supplied by Liaoning Kaiwei Biotechnology Co., Ltd. and consisted mainly of 20% wheat germ, 25% hops extract, 30% grape seed extract and 25% inert carrier (silica). Wheat germ was supplied in powder form. Hops extract contained ≥70% xanthohumol. Grape seed extract contained ≥90% proanthocyanidins.

### Animals and experimental design

The trial was a completely randomised group design. Four hundred and eighty 1-day-old AA + male broilers of similar weight (44.82 ± 0.26) and health were selected and randomly divided into four groups of six replicates each, with 20 broilers in each replicate. The experimental group consisted of a base diet group fed a basic basal diet and a base diet group fed 0.05%, 0.1% and 0.2% of BX.

### Animals feeding management

All birds were housed in the experimental cages (1.25 × 0.80 × 0.50 m/cage) in a test broilers room. The test broilers house was a fully enclosed house with an automatic environmental control system to ensure optimum temperature and humidity (Temperature was started at 33°C and reduced by 3°C every week up to 22°C, and 65% relative humidity). Birds were free to feed and drink. The lighting programme on days 1–7 and 36–42 was 24 h per day throughout the trial period. The lighting programme on days 8–30 provided 20 h per day and 4 h of darkness. After day 31, the darkness hours were gradually reduced. Diets were formulated to meet the nutrient requirements recommend by the National Research Council, (1994) and provided in mash form (Table 1).

**TABLE 1:** Composition and nutrient levels of the basal diet.

Items	Contents	
	Days 1–21	Days 22–42
Ingredients (%)^a^		
Corn	60.4	64.05
Soybean meal	34.4	30
CaHPO_4_	1.40	1.30
CaCO_3_	1.21	1.12
NaCl	0.25	0.25
Soybean oil	1.00	2.00
Choline chloride	0.05	0.05
Lysine	0.08	0.10
*DL*-Met	0.21	0.13
Premix	1	1
Total	100.00	100.00
Nutrient levels (%)^b^		
ME (MJ/Kg)	12.14	12.51
CP	21.17	19.24
Available phosphorus	0.38	0.36
Lys	1.29	1.15
Met	0.67	0.48
Met + Cys	1.00	0.72
Ca	0.92	0.87

^a^ Each kg of premix provides: VA, 5000 IU; VD, 10,000 IU; VE, 75.0 IU; VK_3_, 18.8 mg; VB_1_, 9.8 mg; VB_2_, 28.8 mg; VB_6_, 19.6 mg; VB_12_, 0.1 mg; Biotin, 2.5 mg; Folic Acid, 4.9 mg; d-Pantothenic acid, 58.8 mg; Nicotinic acid, 196.0 mg; Zn, 37.6 mg; Fe, 40.0 mg; Cu, 4.0 mg; Mn, 50.0 mg; I, 0.2 mg; Se, 0.2 mg.

^b^ The nutrient levels were calculated values.

### Test indicator determination

#### Growth performance

Birds were weighed at 1, 21, and 42 days. Feed intake was recorded in replicates throughout the trial and average daily feed intake (ADG), average daily weight gain (ADFI) and meat to feed ratio (F/G) were calculated.

#### Blood indicators

On days 21 and 42 of the trial, 4 ml of blood was collected from the broiler’s lower wing vein, left to stand for 30 min and centrifuged at 1,200 r/min for 15 min to extract the supernatant. The potency of serum antibodies to Newcastle disease and avian influenza H9 is determined by a haemagglutination inhibition test. Serum levels of albumin (ALB), total protein (TP), globulin (GLOB), alanine transaminase (ALT), alkaline phosphatase (ALP), and glucose (GLU) were measured using a fully automated biochemical analyser.

#### Faecal microbiota

On day 21 and 42 of the trial, a 1 g sample of broiler manure from each replicate was collected and transported on ice to the laboratory following the method of Dang et al. (2020). Each replicate of 1 g faecal sample was diluted and mixed with 9 ml of 1% peptone broth. The viable counts of *E. coli*, *Lactobacillus* and *Salmonella* in faecal samples were determined on McConkey agar plates, MRS agar plates and BS agar plates (in a 10 g/L peptide solution) in a biosafety cabinet. The microbial count is ultimately expressed as log_10_ colony forming units per Gram of faeces.

#### Noxious gas emissions

On day 21 and 42 of the trial, fresh broiler manure was collected from each replicate and ammonia and hydrogen sulphide emissions from the manure were determined using the method of Dang et al. (2020). The manure was placed in a 2 L plastic box with small holes attached to the side and fermented at room temperature (25°C) for 12, 24, and 48 h. The air sample is then collected with a gas collection pump from above the small holes on either side. NH_3_ and H_2_S concentrations are measured in the range of 0.00–100.00 mg/m^3^.

### Data analysis

The data was designed using a completely randomised grouping design. Replicate cage serves as the experimental unit. Multiple comparisons of significant differences in means were performed using the one-way ANOVA LSD method in SPSS 25.0 and visualisation was completed using Graphpad Prism 8. Orthogonal contrasts were used to examine the linear and quadratic effects in response to increasing dietary BX levels. Results are expressed as mean and standard deviation, with *p* < 0.05 indicating a significant difference.

## Results

### Growth performance

As shown in Table 2, Dietary supplementation with graded levels of BX linearly increased ADG and ADFI in broilers during days 22–42 days and 1–42 (*p* < 0.05), and there was a quadratic effect on ADG in broilers during days 22–42 and 1–42 (*p* < 0.05). Dietarys supplemented with 0.2% BX significantly increased ADG and ADFI at 22–42 and 1–42 days compared to controls (*p* < 0.05). There was no significant effect of dietary supplementation with BX on F/G in all periods (*p* > 0.05).

**TABLE 2:** Effects of dietary supplementation of Wheat germ, Hops and Grape seed extract mixture (BX) on growth performance in broilers.

Items	Dietary BX levels, %				*p*-Value	
	0	0.05	0.1	0.2	Linear	Quadratic
ADG, g/d						
Days 1–21	41.96 ± 0.50	41.93 ± 0.46	42.04 ± 0.46	42.04 ± 0.46	0.670	0.913
Days 22–42	79.23 ± 2.89^b^	80.29 ± 2.50^ab^	82.30 ± 3.05^ab^	83.16 ± 2.51^a^	0.009	0.035
Days 1–42	60.60 ± 1.60^b^	61.11 ± 1.42^ab^	62.17 ± 1.70^ab^	62.60 ± 1.45^a^	0.016	0.058
ADFI, g/d						
Days 1–21	52.71 ± 0.55	52.57 ± 0.75	52.73 ± 0.43	52.72 ± 0.66	0.874	0.951
Days 22–42	134.48 ± 3.78^b^	136.10 ± 3.81^ab^	138.93 ± 3.70^ab^	139.81 ± 3.84^a^	0.010	0.036
Days 1–42	93.60 ± 2.10^b^	94.34 ± 2.08^ab^	95.83 ± 1.98^ab^	96.27 ± 2.14^a^	0.016	0.058
F/G						
Days 1–21	1.25 ± 0.01	1.26 ± 0.01	1.25 ± 0.01	1.25 ± 0.01	0.748	0.924
Days 22–42	1.70 ± 0.02	1.70 ± 0.02	1.69 ± 0.02	1.68 ± 0.01	0.076	0.214
Days 1–42	1.55 ± 0.01	1.54 ± 0.01	1.54 ± 0.02	1.54 ± 0.01	0.156	0.374

ADG, average daily gain; ADFI, average daily feed intake; F/G feed-to-weight ratio.

^a,b^Means in the same row with different superscripts are significantly different (*p* < 0.05).

### Blood indicators

**FIGURE 1:**
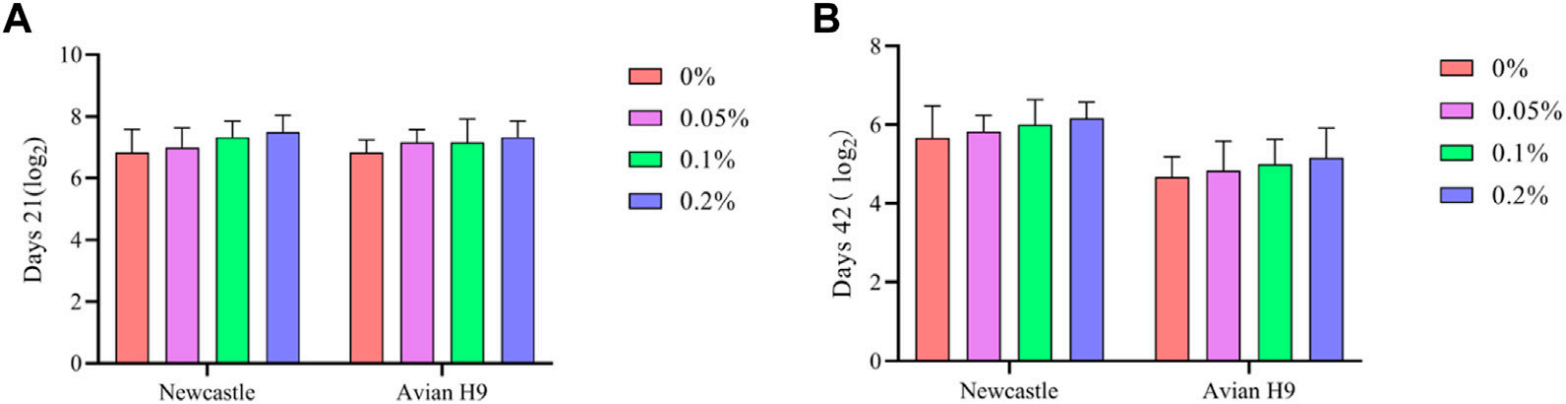
Effects of dietary supplementation of Wheat germ, Hops and Grape seed extract mixture (BX) on serum antibody potency in broilers. **(A)** Newcastle disease and Avian influenza H9 antibody potency in 21 days broiler sera; **(B)** Newcastle disease and Avian influenza H9 antibody potency in 42 days broiler sera.

As shown in Figure 1, Dietary supplementation with graded levels of BX increased the potency of Newcastle disease and avian influenza H9 antibodies in broiler serum at days 21 and 42, but the statistical results did not reach significant levels (*p* > 0.05).

**FIGURE 2:**
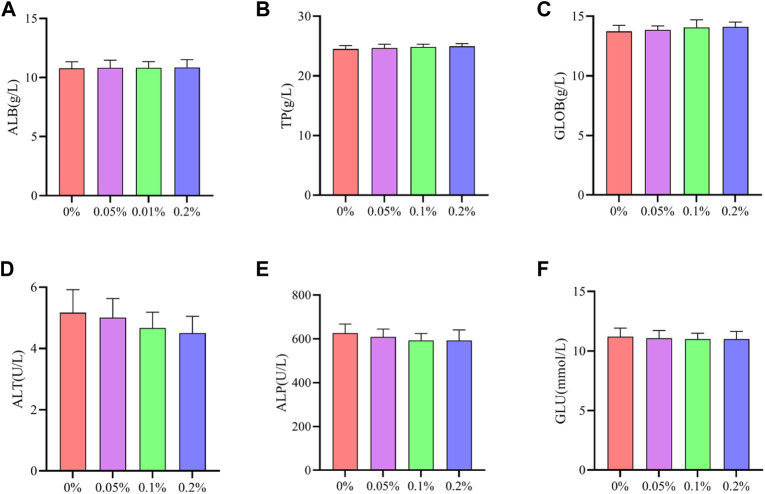
Effects of dietary supplementation of Wheat germ, Hops and Grape seed extract mixture (BX) on serum biochemical parameters in 21 days broilers. **(A)** Albumin; **(B)** Total protein; **(C)** Globulin; **(D)** Alanine transaminase; **(E)** Alkaline phosphatase; **(F)** Glucose.

**FIGURE 3:**
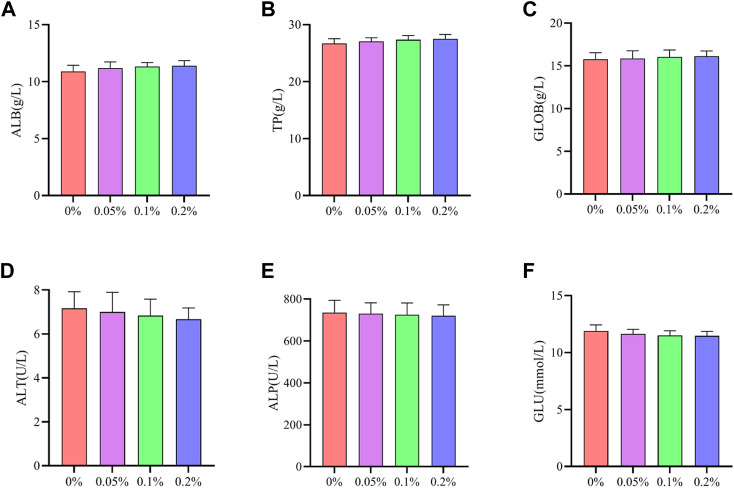
Effects of dietary supplementation of Wheat germ, Hops and Grape seed extract mixture (BX) on serum biochemical parameters in 42 days broilers. **(A)** Albumin; **(B)** Total protein; **(C)** Globulin; **(D)** Alanine transaminase; **(E)** Alkaline phosphatase; **(F)** Glucose.

As shown in Figures 2, 3, Dietary supplementation with graded levels of BX increased broiler serum levels of 21-day GLOB and 42-day ALB, and decreased serum levels of ALT at days 21 and 42, but the statistical results did not reach significant levels (*p* > 0.05).

### Faecal microbiota

**FIGURE 4:**
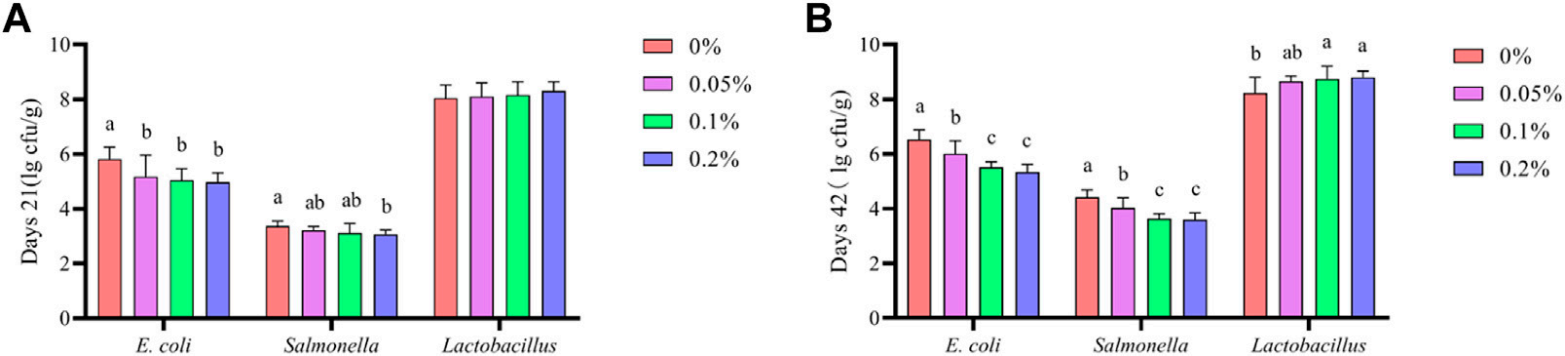
Effects of dietary supplementation of Wheat germ, Hops and Grape seed extract mixture (BX) on faecal microbiota in broilers. **(A)** Days 21; **(B)** Days 42. ^a,b,c,d^Means in the different groups with different superscripts are significantly different (*p* < 0.05). The same as below.

As shown in Figure 4, Dietary supplementation with graded levels of BX significantly reduced *E. coli* levels in broiler faeces at 21 days (*p* < 0.05). *Salmonella* levels in 21-day broiler faeces was significantly reduced when supplemented with 0.2% BX compared to the control group (*p* < 0.05). Dietary supplementation with graded levels of BX significantly reduced *E. coli* and *Salmonella* levels in broiler faeces at 42 days (*p* < 0.05). *Lactobacillus* levels in 42-day broiler faeces was significantly increased relative to the control when supplemented at 0.1% and 0.2% (*p* < 0.05).

### Noxious gas emissions

**FIGURE 5:**
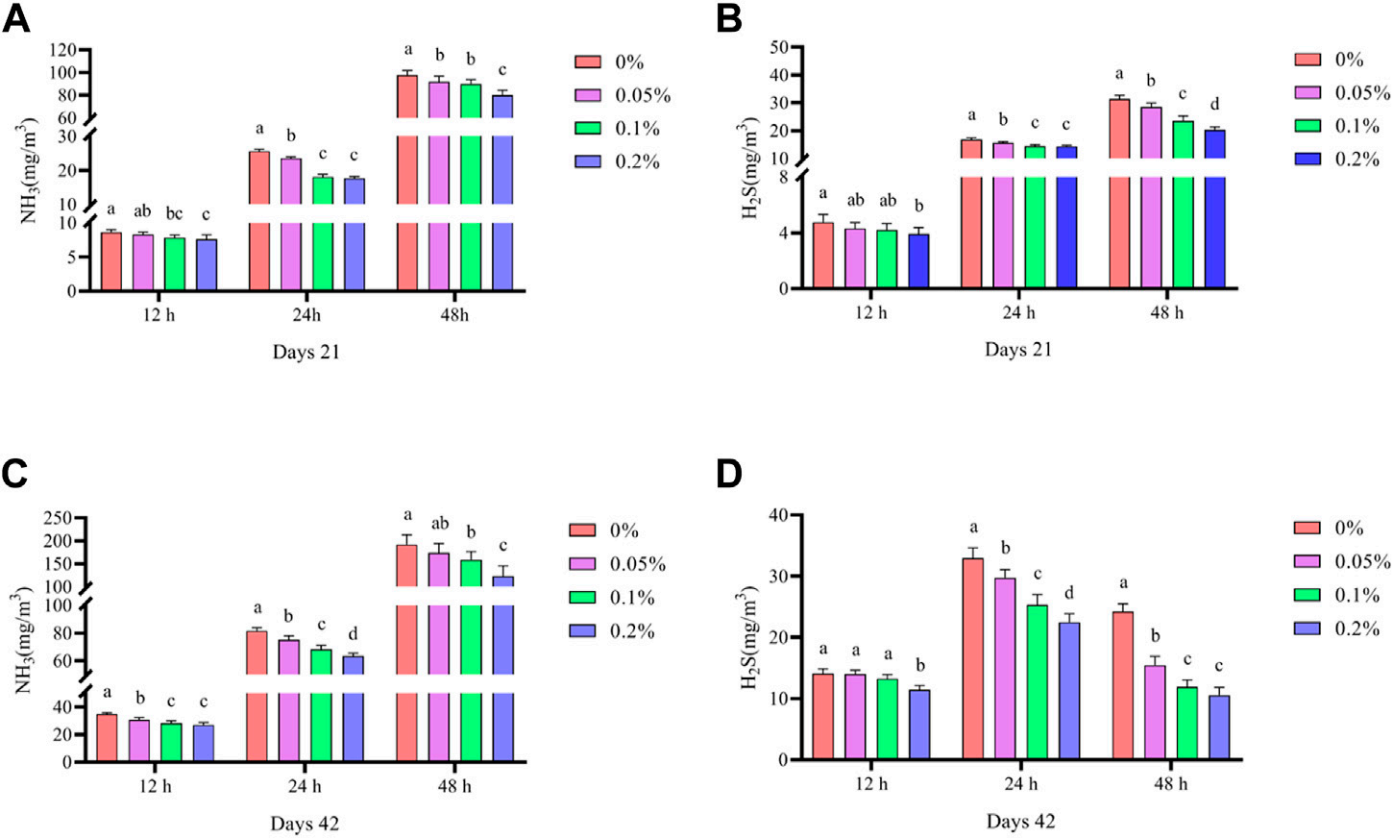
Effects of dietary supplementation of Wheat germ, Hops and Grape seed extract mixture (BX) on noxious gas emissions in broilers. **(A)** Days 21 NH_3_ emissions; **(B)** Days 21 H_2_S emissions; **(C)** Days 42 NH_3_ emissions; **(D)** Days 21 H_2_S emissions.

As shown in Figure 5, Dietary supplementation with graded levels of BX significantly reduced NH_3_ and H_2_S emissions in faeces at days 21 and 42 (*p* < 0.05). In contrast, at 0.2% BX supplementation, NH_3_ and H_2_S emissions were at their lowest values at each fermentation time.

## Discussion

The results of this trial showed that supplementing the broiler diet with wheat germ, hops and grape seed extract mixture (BX) mainly improved the growth performance of AA + broilers in the later stages of production (22–42 days). And as the amount added increases, so does the ADG and ADFI. Although there were few reports on whether wheat germ, hops and grape seed extracts promote broiler growth, some reports suggested positive effects (Abu and Ibrahim, 2018; Goluch et al., 2023). Possible reasons for this were that natural antioxidants protect the intestinal mucosa from oxidative damage and pathogens, while limiting peristaltic activity in digestive disorders, and that some reduction in intestinal motility might lead to better nutrient absorption (Kermauner and Laurenčič, 2008). To date, there had been much evidence that wheat germ, hops and grape seed extracts had antioxidant, antibacterial and anti-inflammatory effects. However, further research is needed to determine whether they promote growth, or whether they do so in other ways.

This pilot study found that although BX increased Newcastle disease antibody potency, ALB and GLOB levels and reduced ALT levels in broiler serum, none of these reached statistically significant levels. The increased potency of Newcastle disease antibodies in serum might be due to the immunostimulatory function of grape seed extract (GSE), mainly due to its antioxidant and free radical scavenging properties, which increased the integrity and added value of B lymphocytes that differentiate into antibody-producing plasma cells (Hamilos et al., 1989). Previous studies had shown that the addition of GSE to the diet could activate the Nrf2 signalling pathway and improved resistance to oxidative stress in broilers (Long et al., 2016; Rajput et al., 2019). Ali Rajput et al. (2017) found that the addition of GSE (250 and 500 mg/kg) to the diet significantly increased serum TP, ALB, and GLOB levels. This is similar to the results of this test.

In this study, the faecal microbiota of broilers were counted at 21 and 42 days and harmful gas emissions were measured. The results showed that dietary supplementation with BX significantly reduced *E. coli* and *Salmonella* counts in broiler faeces. At the same time, the number of *Lactobacillus* increased, suggesting that the addition of BX to the diet could selectively inhibit the growth of pathogens. There was also a significant reduction in NH_3_ and H_2_S emissions from faeces. The antimicrobial activity of BX was attributed to lectins, tannins, flavonoids, *ß*-acids, and xanthohumol. These bioactive components exert their antimicrobial action through different mechanisms that had been reported by most researchers. Studies had confirmed that the flavonoids in GSE could promote the growth of beneficial intestinal bacteria and inhibit certain pathogenic bacteria such as *E. coli*, *Candida albicans*, and *S. aureus* (Silván et al., 2013; Brenes et al., 2016). Cao et al. (2020) reported that the polyphenols in GSE could show positive prebiotic effects by promoting the growth of *Lactobacillus* and *Bifidobacterium* to maintain intestinal health. In addition, lectins from wheat germ had been shown to be viable options for various biomedical and therapeutic applications (Ryva et al., 2019). The *ß*-acid of hops was dipentylated at the C-6 site and also has antibacterial activity (Forino et al., 2016). The results of several studies had shown that faecal odour and ammonia emissions are associated with nutrient utilisation and the gut microbial ecosystem (Misiukiewicz et al., 2021). Along with *Pseudomonas*, *Citrobacter*, *Aeromonas* and *Salmonella*, *E. coli* was identified as the most promising H_2_S producing bacteria (Maker and Washington, 1974). Fecal NH_3_ and H_2_S emissions were also reduced due to the addition of BX to the dietary reducing the number of *E. coli* and *Salmonella* in broiler faeces. Based on the results of this trial, we hypothesize that one of the reasons for the increased growth performance of broilers at days 22–42 due to BX supplementation in the diet is the antioxidant properties of BX. Is this also due to the antibacterial properties of BX? The reduction in harmful bacteria and the increase in beneficial bacteria maintains a healthy gut and further promotes digestion and absorption of the diet in broilers, thus improving growth performance. This conjecture has yet to be tested through subsequent studies.

## Conclusion

Dietary supplementation with Wheat germ, Hops and Grape seeds mixture (BX) improved the growth performance of AA + broilers in the later stages of growth (22–42 days). Dietary supplementation with BX reduced the levels of *E. coli* and *Salmonella* in broiler faeces, as well as the emission of NH_3_ and H_2_S. and lead to increased levels of *Lactobacillus*.

## Data Availability

The raw data supporting the conclusion of this article will be made available by the authors, without undue reservation.
